# Quantification of ocean heat uptake from changes in atmospheric O_2_ and CO_2_ composition

**DOI:** 10.1038/s41598-019-56490-z

**Published:** 2019-12-27

**Authors:** L. Resplandy, R. F. Keeling, Y. Eddebbar, M. Brooks, R. Wang, L. Bopp, M. C. Long, J. P. Dunne, W. Koeve, A. Oschlies

**Affiliations:** 1 Department of Geosciences and Princeton Environmental Institute, Princeton University, Princeton, USA; 2 Scripps Institution of Oceanography, University of California San Diego, La Jolla, USA; 3 Department of Environmental Science and Engineering, Fudan University, Shanghai, 200433 China; 4 Laboratoire de Météorologie Dynamique/Institut Pierre Simon Laplace, CNRS/ENS/X/UPMC, Département de Géosciences, Ecole Normale Supérieure, Paris, France; 5 National Center for Atmospheric Research, Boulder, USA; 6 NOAA, Geophysical Fluid Dynamics Laboratory, Princeton, USA; 7 GEOMAR Helmholtz Centre for Ocean Research, Kiel, Germany

**Keywords:** Ocean sciences, Climate sciences

## Abstract

The ocean is the main source of thermal inertia in the climate system. Ocean heat uptake during recent decades has been quantified using ocean temperature measurements. However, these estimates all use the same imperfect ocean dataset and share additional uncertainty due to sparse coverage, especially before 2007. Here, we provide an independent estimate by using measurements of atmospheric oxygen (O_2_) and carbon dioxide (CO_2_) – levels of which increase as the ocean warms and releases gases – as a whole ocean thermometer. We show that the ocean gained 1.29 ± 0.79 × 10^22^ Joules of heat per year between 1991 and 2016, equivalent to a planetary energy imbalance of 0.80 ± 0.49 W watts per square metre of Earth’s surface. We also find that the ocean-warming effect that led to the outgassing of O_2_ and CO_2_ can be isolated from the direct effects of anthropogenic emissions and CO_2_ sinks. Our result – which relies on high-precision O_2_ atmospheric measurements dating back to 1991 – leverages an integrative Earth system approach and provides much needed independent confirmation of heat uptake estimated from ocean data.

## Introduction

**Figure 1:**
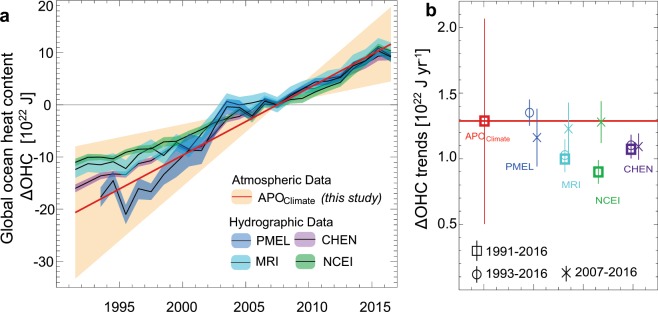
Change in global ocean heat content (ΔOHC). (**a**) ΔOHC derived from hydrographic and atmospheric observations (normalized to zero in 2007, ±1-σ uncertainty). (**b**) Linear least-squares trends for 1991–2016, 1993–2016 and 2007–2016 (±1-σ uncertainty). Hydrography-based ΔOHC estimates combine warming rates at ocean depths of 0 to 2,000 m warming rates (Cheng and co-authors (CHEN)^8^, Pacific Marine Environmental Laboratory (PMEL)^6^, Meteorological Research Institute (MRI)^5^ and National Center for Environmental Information (NCEI)^47^) with revised deep ocean (depth of more than 2000 m) of ref. ^7^ (Tables S1, S2). The atmospheric-based estimate which uses observed atmospheric potential oxygen trends (ΔAPO_Climate_) and model-based ΔAPO_Climate_-to-ΔOHC ratio, does not resolve interannual variations.

A fundamental measure of global warming is the heat uptake by the ocean, which represents more than 90% of the excess energy gained by the Earth^1^. This ocean warming has been quantified using hydrographic temperature measurements, including data from the Argo float program, which expanded coverage after 2007^2–4^. As shown in Fig. 1, the most recent temperature-based estimates of ocean warming^5–8^ show good agreement for 2007-2016 (1.09 ± 0.10 to 1.16 ± 0.2 × 10^22^ J yr^−1^), but a larger spread when extending back to include the sparser 1990s data (0.90 ± 0.09 to 1.36 ± 0.10 × 10^22^ J yr^−1^ for 1993–2015). The spread is mostly caused by gap-filling methods and systematic errors^5,8,9^, which together introduce uncertainties up to 25–50% in warming trends^10^. Because temperature-based estimates use the same upper-ocean observations and linear warming trend for depths below 2000 m (ref. ^6^.), they may share additional unknown systematic errors^8^. An alternative method based on the top of the atmosphere energy balance^11^ is also not truly independent, because it is subject to large systematic errors when estimating long-term trends and therefore depends on the same hydrographic measurements for calibration^11–14^. Here we introduce a third method, based on changes in the abundances of gases in the atmosphere, which respond to whole-ocean warming through the temperature dependence of gas solubility in seawater. This method is not limited by data sparseness, because fast mixing in the atmosphere efficiently integrates the global ocean signal.

Changes in ocean heat content on seasonal^15^ and glacial-interglacial^16^ time-scales have been reconstructed using measurements of noble gases in modern or ancient air. Our method is similar, but instead of relying on noble gases (e.g. Ar/N_2_), which lack sufficient accuracy as yet^15^, we rely on measurements of atmospheric O_2_ and CO_2_, which can be summed to yield a tracer “atmospheric potential oxygen” (APO) that responds to warming similar to a noble gas^17^. When the ocean warms, the solubility of O_2_ and CO_2_ drops, and the amount of gas lost by the ocean can be quantified with the complementary change observed in the atmosphere. Precise atmospheric O_2_ measurements began in 1991 (CO_2_ in 1958), enabling APO-based ocean heat content reconstructions that span nearly three decades^18^.

## Results

### Atmospheric potential oxygen trend and components

**Figure 2:**
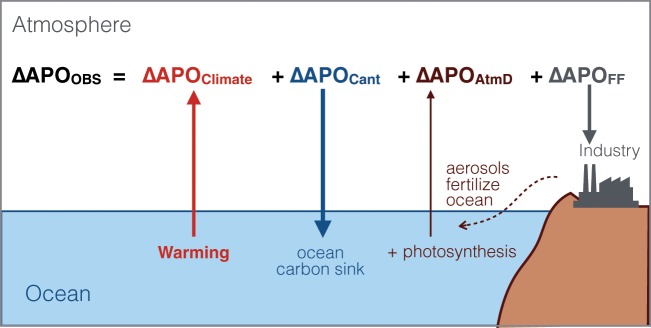
Processes contributing to observed changes in atmospheric potential oxygen (ΔAPO_OBS_). Industrial processes (fossil fuel burning and cement production, ΔAPO_FF_) and ocean sink for anthropogenic carbon (ΔAPO_Cant_) remove APO from the atmosphere. The fertilization effect of anthropogenic aerosol deposition which promotes marine photosynthesis (ΔAPO_AtmD_) and the changes in solubility, biology and ocean circulation due to warming (ΔAPO_Climate_) release APO into the atmosphere. This study shows that ΔAPO_Climate_ can be used to estimate long term changes in global ocean warming.

APO (=O_2_ + 1.05 × CO_2_) is computed using observed atmospheric O_2_/N_2_ mole ratios and CO_2_ mole fractions (see Methods)^18–20^. By design, APO is insensitive to exchanges with land ecosystems, which produce changes in O_2_ and CO_2_ that largely cancel in APO owing to their approximate 1.05 O_2_:C oxidative ratio. Time-series measurements at remote sites show a global long-term decline in APO ΔAPO_OBS_ = 255.93 ± 13.74 per meg (units defined in Methods) between 1991 and 2016. ΔAPO_OBS_ is driven by four primary contributors, illustrated in Fig. 2:

1 $${\Delta {\rm{APO}}}_{{\rm{OBS}}}={\Delta {\rm{APO}}}_{{\rm{FF}}}+{\Delta {\rm{APO}}}_{{\rm{Cant}}}+{\Delta {\rm{APO}}}_{{\rm{AtmD}}}+{\Delta {\rm{APO}}}_{{\rm{Climate}}}$$

where ΔAPO_FF_ is the decrease in APO caused by industrial processes (fossil fuel burning and cement production) which in aggregate consume more than 1.05 moles of O_2_ for each mole of CO_2_ released; ΔAPO_Cant_ accounts for the oceanic uptake of excess anthropogenic atmospheric CO_2_; APO_AtmD_ accounts for for air-sea exchanges driven by ocean fertilization from anthropogenic aerosol deposition (increased fertilization leads to increased photosynthesis, with a concomitant release of O_2_ and uptake of CO_2_); and ΔAPO_Climate_ accounts for air-sea fluxes of O_2_, CO_2_ and N_2_ driven by ocean processes, including warming-induced changes in solubility, in ocean circulation, and in photosynthesis and respiration (N_2_ influences O_2_/N_2_ ratios). Here, we derive ΔAPO_Climate_ from Eq. (1) and show that it tracks ocean warming.

**Figure 3:**
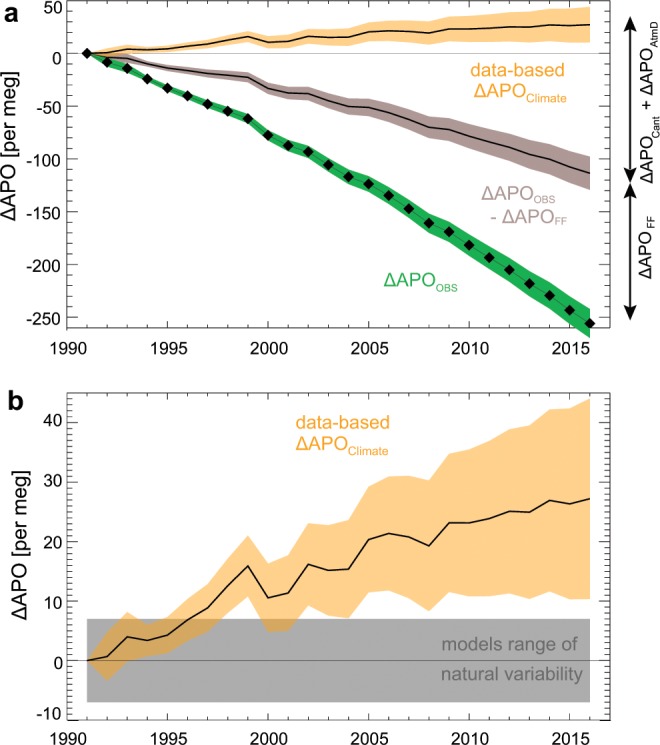
Data-based estimates of global ΔAPO_Climate_. (**a**) ΔAPO_Climate_ estimated from observed APO (ΔAPO_OBS_) from the Scripps Institution of Oceanography network (1991–2016), and corrected from fossil fuel burning, ocean anthropogenic carbon uptake and anthropogenic aerosols deposition (ΔAPO_Climate_ = ΔAPO_OBS_ –ΔAPO_FF_ –ΔAPO_Cant_ –ΔAPO_AtmD_) and their 1σ uncertainty ranges. (**b**) The increase in global ΔAPO_Climate_ (±1σ interval) exceeds the range of 26-year trends expected from natural variations in four earth system models (CESM, GFDL, IPSL and UVic, shown in grey). Uncertainties and contributions to ΔAPO_Climate_ are given in Tables S3, S4 and S6.

We estimate ΔAPO_FF_ using fossil fuel and cement inventories^21^, finding ΔAPO_FF_ = −142.38 ± 7.65 per meg (Fig. 3). ΔAPO_Cant_ is controlled by the increase in atmospheric CO_2_ and by ocean mixing, which is quantified by transient tracers distributions including chlorofluocarbons (CFCs)^22^; we find that ΔAPO_Cant_ = −147.75 ± 3.69 per meg. ΔAPO_Cant_ is relatively precise because it excludes the effect of changing ocean biology and circulation on natural carbon fluxes that are included in ΔAPO_Climate_. ΔAPO_AtmD_ is derived from ocean model simulations with and without aerosols fertilization (phosphate, iron and nitrogen; Fig. S1)^23^. ΔAPO_AtmD_ is uncertain, owing in part to uncertainties in iron availability to photosynthetic organisms, but is relatively small compared with the other terms: ΔAPO_AtmD_ = 7.00 ± 3.50 per meg. From Eq. (1), we therefore find ΔAPO_Climate_ = 27.21 ± 16.85 per meg, corresponding to a least-squares linear trend of +1.11 ± 0.68 per meg per year – larger than the trends expected from 26-year natural variations alone in four earth system models (the Community Earth System Model (CESM) and the Geophysical Fluid Dynamics Laboratory (GFDL), Institut Pierre Simon Laplace (IPSL) and University of Victoria (UVic) models). As show in Fig. 3, a clear increase in ΔAPO_Climate_ emerges over the period January 1991 to the end of December 2016.

### Linking atmospheric potential oxygen to ocean warming

**Figure 4:**
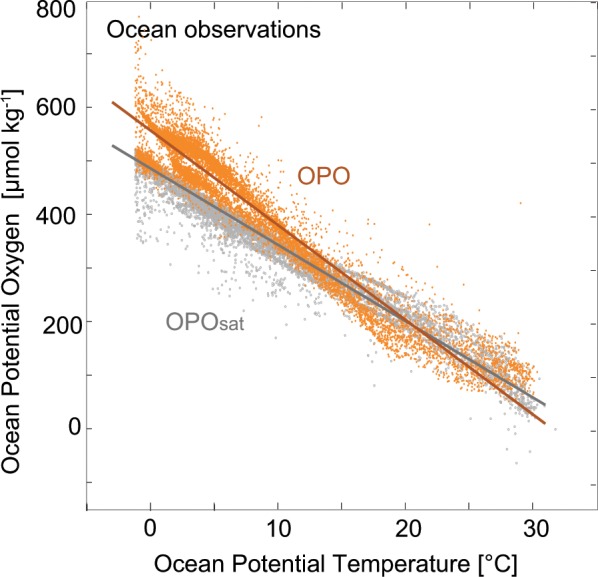
Observed link between potential oxygen and ocean heat. OPO concentrations *in-situ* (OPO, yellow) and at saturation based on O_2_ and CO_2_ solubility (OPO_sat_, grey) as a function of ocean temperature in the GLODAPv2 database^49^.

A starting point for understanding ΔAPO_Climate_ is to imagine that O_2_ and CO_2_ behave like inert gases, such that the air-sea fluxes are dominated by temperature-driven solubility changes. In this case, APO would increase by around 0.8 per meg per 10^22^ J of warming, with O_2_ and CO_2_ solubility changes accounting for an increase of +1.0 per meg per 10^22^ J partly offset by the N_2_ contribution of −0.2 per meg per 10^22^ J (Methods). Support for the dominance of solubility in ΔAPO_Climate_ can be found in the natural distribution of O_2_ and carbon in the ocean. Ocean potential oxygen (OPO) is a dissolved tracer that mirrors APO_Climate_ and tracks changes in air-sea O_2_ and CO_2_ fluxes^17^. Observed OPO abundance is strongly tied to ocean potential temperature (Fig. 4): warming induces OPO loss and cooling induces OPO gain. The OPO-to-temperature trend of −4.43 nmol J^−1^ is within 18% of the trend of −3.65 nmol J^−1^ expected from solubility alone (OPO_sat_-to-temperature). Biological effects (related to changes in ocean circulation and photosynthesis/respiration) on CO_2_ and O_2_ substantially cancel in OPO (Fig. S2), while thermal impacts reinforce each other, with warming waters releasing both O_2_ and CO_2_ to the atmosphere and increasing ΔAPO_Climate_.

Further support for the dominance of solubility on ΔAPO_Climate_ is found on multidecadal timescales in four Earth system models mentioned above, which yield OPO-to-temperature ratios between −4.69 and −4.36 nmol J^−1^, bracketing the ratio of −4.43 nmol J^−1^ found in hydrographic observations (Fig. S3). The models also simulate a very close relationship between ΔAPO_Climate_ and the change in global ocean heat content (ΔOHC) that occurs during the simulations (1920–2100), with an atmospheric build-up in APO between 0.82 and 0.98 per meg per 10^22^ J (Figs. S3 and S4) – close to the ratio expected from temperature-driven solubility changes alone (0.8 per meg per 10^22^ J). By dividing the simulated APO change into separate biological and thermal components, we show that solubility changes account for more than 80% of ΔAPO_Climate_, while biologically driven changes account for 5% to 20% (Fig. S4). This partitioning found in response to transient warming is very similar to the partitioning found in hydrographic data (where solubility and biology contribute 82% and 18% respectively, to the OPO-to-temperature ratio, Fig. 4).

Small differences between individual model ΔAPO_Climate_-to-ΔOHC relationships (0.82 to 0.98 per meg per 10^22^ J) reflect systematic differences in biological fluxes. Models with stronger biological effects (IPSL and UVic) yield stronger oceanic loss of OPO and stronger release of APO for a given ocean warming (more negative OPO-to-temperature and higher ∆APO_Climate_-to-∆OHC, Fig. S3b). Using this relationship, we find that a ∆APO_Climate_-to-∆OHC ratio of 0.86 ± 0.03 per meg per 10^22^ J is compatible with observed OPO-to-temperature ratio. Combining this constrained ΔAPO_Climate_-to-ΔOHC ratio (0.86 ± 0.03 per meg per 10^22^ J) with the observation-based trend in ΔAPO_Climate_ (1.11 ± 0.68 per meg/y), yields a warming trend of 1.29 ± 0.79 × 10^22^ J yr^−1^ between 1991 and 2016. As shown in Fig. 1, this APO-based estimate of ocean heat uptake is centered near the high end of recent ocean-based estimates, while agreeing with all estimates to within the uncertainties. In our estimate, the largest single source of uncertainty is the scale error from the span calibration of the O_2_/N_2_ analyzer (Table S6), which can be reduced via within-lab and inter-lab comparisons. The uncertainty in the estimated heat uptake since 1991 should therefore be significantly reduced in future updates.

## Discussion

Most recent ocean-based estimates of warming suggest an increase relative to prior estimates^1,5,9^. The independent APO estimate is fully in line with this upwards revision, although uncertainties are large. Our central value of ΔOHC would call for a steric sea level rise of 1.53 mm y^−1^ (see Methods), also in agreement with satellite-derived constraints on thermal expansion, corrected for the fresh water contribution (1.50 ± 0.40 mm y^−1^)^24,25^. Our result suggests that the ocean contributes 0.80 ± 0.49 W/m^2^ to the Earth energy imbalance over the 1991–2016 period (Earth surface of 5.1 × 10 ^14^ m^2^), with implications for equilibrium climate sensitivity and the climate system lagged response to anthropogenic forcing^26,27^.

We find that the APO-heat coupling (APO_Climate_) is most robust on decadal and longer time-scales. Strong cancelation of biological O_2_ and CO_2_ fluxes is not expected on all temporal scales. On seasonal time-scales, air-sea O_2_ fluxes driven by marine photosynthesis are ~8 times larger than those of CO_2_ due to slow equilibration of CO_2_^28^. More complex coupling is also possible on interannual time-scales^29^, such as the weaker lagged air-sea CO_2_ flux compared to O_2_ during El Nino events^30^.

Atmospheric O_2_ and CO_2_ measurements have previously been applied to estimate global land and ocean CO_2_ sinks, but relied on ocean heat content estimates and model-based oceanic O_2_-to-heat ratios to correct for climate-driven O_2_ outgassing^31–33^. Here we have reversed this logic, using estimates of other quantities to constrain the ocean heating. Our approach exploits the APO-heat relationship, which is stronger than the O_2_-heat relationship (See Methods for estimate of climate-driven ocean O_2_ outgassing based on our results). Further work to constrain the separate contributions of O_2_ and CO_2_ to APO is needed to refine estimates of ocean and land carbon sinks using atmospheric O_2_.

The results presented here were previously published in *Nature*, 563, 105–108 (2018)^34^. The paper was subsequently retracted due to the underestimation of the uncertainties. Certain systematic errors were treated as random errors, and the uncertainty in the land oxidative ratio on the APO budget was neglected. These issues have been corrected in the current paper. The correction did not substantially change the central estimate of ocean warming but led to a roughly fourfold increase in uncertainties.

## Methods

### Observed changes in atmospheric potential oxygen (ΔAPO_OBS_)

A change in atmospheric potential oxygen concentration (in per meg) is defined following^19^:

$$\Delta (\delta {\rm{A}}{\rm{P}}{\rm{O}})[{\rm{p}}{\rm{e}}{\rm{r}}\,{\rm{m}}{\rm{e}}{\rm{g}}]=\Delta ({\delta {\rm{O}}}_{2}/{{\rm{N}}}_{2})+\frac{{\alpha }_{{\rm{B}}}}{{{\rm{X}}}_{{\rm{O}}2}}\cdot ({{\rm{X}}}_{{\rm{C}}{\rm{O}}2}-350)$$

with

$$({{\rm{\delta }}{\rm{O}}}_{2}/{{\rm{N}}}_{2})[{\rm{per}}\,{\rm{meg}}]=\frac{{{\rm{O}}}_{2}/{{\rm{N}}}_{2}({\rm{sample}})}{{{\rm{O}}}_{2}/{{\rm{N}}}_{2}({\rm{reference}})}\,-\,1$$

where Δ(δO_2_/N_2_) is the atmospheric change in δO_2_/N_2_ ratios (in per meg), X_CO2_ is the CO_2_ concentration in the air parcel (in ppm, i.e. μmol mol^−1^) and 350 is an arbitrary reference, α_B_ (=1.05) is the approximate O_2_:CO_2_ ratio of terrestrial ecosystems^20^, and X_O2_ (=0.2094) is the reference value of atmospheric mole fraction of O_2_ necessary to convert X_CO2_ from ppm to per meg units. We use an oxidative ratio α_B_ of 1.05 rather than 1.1, following the recommendation of ref. ^20^. who argue that woody biomass, which has an oxidative ratio near 1.05, likely dominates the long-term carbon sources and sinks on land.

ΔAPO_OBS_ is computed from *in situ* atmospheric changes in CO_2_ concentrations and O_2_/N_2_ ratios^20^ measured at stations of the Scripps Institution of Oceanography network (available online at http://scrippso2.ucsd.edu)^18,35^. The global average ΔAPO_OBS_ is based on a weighted average of the three stations with the longest record (1991 to 2016), i.e. La Jolla (32.9°N, 117°W), Alert (82.5°N, 62.5°W) and Cape Grim (40.5°S, 144.5°E), with weights of 0.25, 0.25 and 0.5 respectively^36^. Station’s annual means are based on bi-monthly data fit to a four-harmonic seasonal cycle and a stiff long-term trend^18^. The uncertainty on ΔAPO_OBS_ was computed by generating 10^6^ time-series with a noise scaled to the random and systematic errors of APO data detailed in Table S3. The uncertainty is taken as the 1-sigma interval (±1 standard deviation) from these 10^6^ realizations (Fig. 3). To assess whether the weighted average of the 3 stations accurately reflects the global trend, we also compared other combinations of stations from 1999 onward when we have reliable data from 9 stations. The differences were consistently smaller than 0.05 per meg yr^−1^ which is negligible compared to the other combined measurement uncertainties.

### Fossil fuel burning and cement production influence on APO (ΔAPO_FF_)

ΔAPO_FF_ is estimated using annual CO_2_ emissions from oil, coal, gas, flaring and cement production (ΔCO_2(i)_ in moles)^21^ weighted by their O_2_:C combustion ratios R_i_^18^:

$${\Delta {\rm{APO}}}_{{\rm{FF}}}[{\rm{per}}\,{\rm{meg}}]=\sum _{{\rm{i}}}\,\frac{{{\rm{\alpha }}}_{{\rm{B}}}-{{\rm{R}}}_{{\rm{i}}}}{{{\rm{X}}}_{{\rm{O}}2}}\times \frac{{\Delta {\rm{CO}}}_{2({\rm{i}})}}{{{\rm{M}}}_{{\rm{air}}}}$$

where M_air_ is the number of moles of dry air in the atmosphere (convert moles of CO_2_ to ppm).

The uncertainty on ΔAPO_FF_ includes uncertainties in CO_2_ emissions (ΔCO_2(i)_)^37^ and in combustion ratios (R_i_ in Tab S3)^18^. Uncertainties in ΔCO_2(i)_ and R_i_ are considered not independent in time and were estimated using an autoregressive model with a correlation time-scale of 20 years following the method of ref. ^38^ (1000 realizations using Monte-Carlo approach). The uncertainty on ΔAPO_FF_ was then estimated by combining the 1000 realizations of ΔCO_2(i)_ and the 1000 realizations of R_i_, yielding a set of 10^6^ estimates of ΔAPO_FF_.

### Ocean anthropogenic carbon uptake influence on APO (ΔAPO_Cant_)

We can divide the ocean CO_2_ uptake (ΔCO_2_) as the sum of three contributions:

$${\Delta {\rm{C}}{\rm{O}}}_{2}={\Delta {\rm{C}}{\rm{a}}{\rm{n}}{\rm{t}}}_{0}+\Delta {\rm{C}}{\rm{a}}{\rm{n}}{\rm{t}}^{\prime} +{\Delta {\rm{C}}{\rm{O}}}_{2{\rm{C}}{\rm{l}}{\rm{i}}{\rm{m}}{\rm{a}}{\rm{t}}{\rm{e}}}\,({\rm{S}}1)$$

where ΔCant_0_ is the flux driven by the rise in CO_2_ assuming steady ocean circulation (ΔCant_0_ is negative, corresponding to uptake by the ocean), ΔCO_2Climate_ is the flux driven by the action of climate on natural carbon in the ocean (ΔCO_2Climate_ is positive, i.e. warming reduces the uptake of natural carbon), and ΔCant’ is the remainder, which accounts for impact of circulation changes on the uptake of carbon driven by rising CO_2_ (ΔCant’ is positive, i.e. warming reduces the uptake of C_ant_). ΔAPO_Cant_ can be expressed as the weighted sum of the two terms ΔCant_0_ and ΔCant’:

$${\Delta {\rm{APO}}}_{{\rm{Cant}}}[{\rm{per}}\,{\rm{meg}}]=\frac{{{\rm{\alpha }}}_{{\rm{B}}}}{{{\rm{X}}}_{{\rm{O}}2}\times {{\rm{M}}}_{{\rm{air}}}}\times {(\Delta {\rm{Cant}}}_{0}+\Delta {\rm{Can}}{\rm{t}}^{\prime} )$$

where ΔCant_0_ and ΔCant’ are in moles. Note that ΔCO_2Climate_ is accounted for in ΔAPO_Climate_.

ΔCant_0_ is taken from the recent ocean inversion scheme with assimilation of observed potential temperature, salinity, radiocarbon, and CFC-11 of ref. ^22^ updated to 2016. ΔCant’ cannot be derived from observations and was estimated to be 0.05 PgC y^−1^, equivalent to a trend of +0.11 per meg^−1^, using model simulations (see details in section Model-based ΔCant’ below).

The uncertainty on ΔAPO_Cant_ is related to uncertainties in ΔCant_0_ and ΔCant’. We allow for uncertainty in ΔCant_0_ following ref. ^22^ using the 10 sensitivity experiments (on ocean vertical and isopycnal diffusivities, data constraint, gas exchange coefficient etc.) available for the ocean inversion and an estimate of the interannual variability in the ocean sink of a 0.2 PgC y^−1^. We also allow an additional 1% uncertainty (<0.03 PgC/y) in ΔCant_0_ due to imperfectly known atmospheric CO_2_ history^39^, taking account of sensitivity to start date (1765 vs 1791), to degree of temporal smoothing, and to using different versions of the record since 1958 (Mauna Loa record versus average of Mauna Loa and South Pole records). This estimate used a variant of the box-diffusion model^40^, and CO_2_ data from ref. ^41^ and the Scripps CO_2_ program (10.6075/J0542KSG). Uncertainties on ΔCant’ are assumed to be 100% of the model-based estimate of ΔCant’ (see details in section Model-based ΔCant’ below).

### Ocean fertilization and atmospheric deposition of anthropogenic aerosol (ΔAPO_AtmD_)

Deposition of anthropogenic aerosol from fossil fuel, biomass burning etc. fertilizes the ocean with nutrients, increases surface photosynthesis and sub-surface respiration^42–44^. The effect of aerosol fertilization is partly counterbalanced by biological processes such as a decline in nitrogen fixation, which would be immediate, and an increase in denitrification in the water column, which would be on time-scales of several 100 years^45^. Fixed anthropogenic nitrogen also fertilizes the land biosphere and coastal oceans by river runoffs, but in these cases, efficient denitrification returns fixed nitrogen to the atmosphere and has little impact on the APO budget on the decadal timescales considered here. The impact of anthropogenic aerosol on O_2_, CO_2_ and APO air-sea fluxes is evaluated with the IPSL ocean model NEMO-PISCES v2^46^ using the difference between simulations with aerosols and a simulation in which the aerosol deposition is fixed to a constant pre-industrial value (equivalent to year 1850, Fig. S1)^23^. We use four simulations with varying aerosols: one includes the combined effect of nitrogen (N), iron (Fe) and phosphorus (P) aerosol deposition, whereas the 3 others only include their individual contribution (N-only, Fe-only or P-only, Fig. S1 and Table S5). Uncertainties at 1-sigma level on ΔAPO_AtmD_ are assumed to be ±50%. See Table S4.

Combined, N, Fe and P deposition accounts for an O_2_ outgassing of 19.0 Tmol y^−1^ for the 1980/2007 period (16 Tmol y^−1^ for entire 1960/2007 simulation period) and an oceanic CO_2_ uptake of 8.3 Tmoly^−1^ for the 1980–2007 period (6.8 Tmo ly^−1^ for entire 1960–2007 simulation period, Fig. S1 and Table S5). The overall impact is ΔAPO_AtmD_ = + 0.28 per meg y^−1^ over 27 years of simulation (1980 to 2007), which we extrapolate to our 1991 to 2016 period. Increased O_2_ outgassing accounts for an increase in APO of +0.51 per meg y^−1^, and CO_2_ uptake accounts for a change in APO of −0.23 per meg y^−1^ (APO_AtmD(O2)_ and APO_AtmD(CO2)_ in Table S3).

The overall effect of N, Fe and P is smaller than the sum of the individual effects (Fig. S1), because of the interplay between the aerosol deposition pattern and nutrient co-limitations in the ocean. Phytoplankton growth in the ocean depends on the availability of the most limiting nutrient. While more available N will promote photosynthesis in regions where N is limiting (for example the tropical Atlantic Ocean), the effect is negligible in regions where Fe, P or any other nutrient are limiting (for example the Southern Ocean) (see Fig. 2 in ref. ^23^).

To our knowledge this is the first estimate of the impact of anthropogenic aerosol deposition on both O_2_ and CO_2_ air-sea fluxes at the global scale. Note however that ref. ^18^ used anthropogenic aerosol N inventories and scaling arguments to estimate an ocean O_2_ loss due to anthropogenic N-deposition only of ~10 ± 10 Tmol y^−1^, slightly weaker than our model estimate of 15.5 Tmol y^−1^.

### ΔAPOClimate trends and uncertainty analysis

We compute the APO response to climate change (ΔAPO_Climate_) via

$${\Delta {\rm{APO}}}_{{\rm{Climate}}}={\Delta {\rm{APO}}}_{{\rm{OBS}}}-{\Delta {\rm{APO}}}_{{\rm{FF}}}-{\Delta {\rm{APO}}}_{{\rm{Cant}}}-{\Delta {\rm{APO}}}_{{\rm{AtmD}}}$$

We combine the estimates of ΔAPO_FF_, ΔAPO_Cant_ and ΔAPO_AtmD_ plus estimates of the contribution from variations in the oxidative ratio to obtain 10^6^ time-series of ΔAPO_FF_ + ΔAPO_Cant_ + ΔAPO_AtmD_ and obtain 10^6^ time-series of ΔAPO_Climate_ using the 10^6^ time-series of ΔAPO_OBS_. We computed the ΔAPO_Climate_ trend and its uncertainty based on the distribution of the unweighted least square fits to each of the 10^6^ ensemble realizations of ΔAPO_Climate_ generated by combining all sources of uncertainty. We find a ΔAPO_Climate_ trend of 1.11 ± 0.68 per meg yr^−1^ for 1991–2016. The individual contributions to the uncertainty are shown in Table S6.

### Hydrography-based estimates of ocean heat uptake (ΔOHC)

We used four global-ocean estimates of ΔOHC based on hydrographic measurements in Fig. 1. Surface to 2000 m warming rates are from ref. ^6^ (PMEL), ref. ^5^ (MRI, climate.mri-jma.go.jp/pub/ocean/ts/v7.2/), an updated version of ref. ^47^ (NCEI, www.nodc.noaa.gov/OC5/3M_HEAT_CONTENT/basin_avt_data.html), and ref. ^8^ (CHEN, http://159.226.119.60/cheng/images_files/TOA_OHC_errorbar_1940_2015_2.txt), with the revised deep ocean (depth >2000 m) constant linear warming rate of 0.10 ± 0.03 × 10^22^ J yr^−1^ of ref. ^7^ based on the global ship-based sections program (GO-SHIP; http://www.go-ship.org)^48^.

### Ocean observations of ocean potential oxygen (OPO)

We used *in-situ* ocean observations from GLODAPv2^49^ combined with an anthropogenic carbon estimate^22^ interpolated at the location of each sample to compute 78,456 values (Glodapv2 quality control = 0, marginal seas and coastal waters were removed) of Oceanic Potential Oxygen (OPO)^17^ via

$${\rm{O}}{\rm{P}}{\rm{O}}={{\rm{O}}}_{2}+{\alpha }_{{\rm{B}}}\times {{\rm{C}}}_{{\rm{p}}{\rm{i}}}^{\ast }$$

where O_2_* and C_pi_* are the ocean conservative tracers related to air-sea fluxes of O_2_ and pre-industrial carbon^50^, and α_B_ is the terrestrial oxidative ratio (α_B_ = 1.05). The thermal component (solubility-driven) of OPO (OPO_sat_) is computed as:

$${{\rm{OPO}}}_{{\rm{sat}}}={{\rm{O}}}_{2{\rm{sat}}}+{{\rm{\alpha }}}_{{\rm{B}}}\times {{\rm{C}}}_{{\rm{pisat}}}$$

where O_2sat_ is the dissolved O_2_ concentration at saturation with the observed temperature and salinity^51^ and C_pisat_ is the dissolved inorganic carbon concentration expected at the observed temperature and salinity, and assuming equilibrium with a pre-industrial partial pressure of CO_2_ of 280 ppm and using pre-formed alkalinity^52^.

### Solubility-driven changes in oceanic and atmospheric potential oxygen

Figure S2 shows a tight and quasi-linear link between observed OPO and potential temperature (−4.43 nmol/J, r^2^ = 0.95), similar to the link found between OPO_sat_ and potential temperature (−3.65 nmol/J, r^2^ = 0.93). This suggests that changes in OPO and hence ΔAPO_Climate_ are driven primarily by changes in thermal air-sea fluxes. In these observations, departures of dissolved oxygen and carbon concentrations (O_2_* and C_pi_*) from their respective saturation curves (O_2sat_ and C_pisat_) due to biological activity tend to balance (Fig. S2). By contrast, thermal effects reinforce each other (O_2sat_ and C_pisat_ both decrease with increasing temperature) and biological effects compensate each other (O_2_* > O_2sat_ and C_pi_* < C_pisat_).

Changes in APO expected from changes in gas solubility in the ocean is an increase of 2.95 nmol per J of warming, which includes the outgassing of O_2_ and CO_2_ following OPO_sat_ (3.65 nmol/J) and the release of of N_2_ (0.6 nmol/J) (Fig. S2b). A change of 2.95 nmol per J of warming is equivalent to an increase of 0.8 per meg/10^22^ J of APO in the atmosphere (=(2.95 × 10^−9^)/(3.7 × 10^19^) × 10^22^ = 1.0 × 10^−6^ = 0.8 per meg per 10^22^ J, with 3.7 × 10^19^ the number of moles of O_2_ in the atmosphere). O_2_ and CO_2_ solubility alone yield an increase in APO of 1.2 per meg/10^22^ J, which is partly counterbalanced by the outgassing of N_2_ that decreases APO by 0.2 per meg/10^22^ J (via the increase of the O_2_/N_2_ ratio).

### Earth system model experiments

We used 4 Earth-system models (ESMs): the Geophysical Fluid Dynamics Laboratory Earth System Models with a nominally level vertical coordinate version GFDL-ESM2M (called GFDL here)^53,54^, the Institut Pierre-Simon Laplace Coupled Model 5 version IPSL-CM5A-LR (IPSL here)^55^, the Community Earth System Model large ensemble CESM-LE (CESM here)^56^ and the UVic model version 2.9 (UVic here)^57^. Evaluation of these models and their biogeochemical components can be found in previous studies^54,56,58–60^. GFDL, IPSL and UVic participated in the Coupled Model Intercomparison Project Phase 5 (CMIP5)^61^.

For GFDL, IPSL and UVic, we used the CMIP5 business as usual “historical-RCP8.5” scenario, the feedback experiment “esmFdbk3” that only includes warming-driven changes associated with anthropogenic emissions (e.g. radiation effects) and the fixed-climate experiment “esmFixClim3” that only includes the direct biogeochemical effects of increasing atmospheric CO_2_ (e.g. uptake of anthropogenic carbon, acidification etc.). For CESM, we also used the historical and RCP8.5 experiments and the separation between anthropogenic carbon from the natural carbon available in this model (carbon tracer separation approach). The feedback approach used for GFDL, IPSL and UVic removes all direct biogeochemical effects of rising atmospheric CO_2_ on the air-sea O_2_ and CO_2_ exchanges, whereas the natural carbon tracer separation approach used for CESM still includes the biogeochemical impacts of increasing atmospheric CO_2_ on the carbon cycle (e.g. acidification) even while it excludes the anthropogenic carbon itself. However, we expect this effect to be small and negligible on our results.

We also used the multicentury preindustrial control simulation “piControl” with no increase in atmospheric CO_2_ to correct for model drift and to estimate the natural internal variability of ΔAPO_Climate_ (Fig. 2). We used model results over the 1920–2100 period, which were available for the four models.

Model OPO was computed as in the observations. Note that for CESM we removed subsurface regions of high denitrification in the eastern equatorial Pacific and Bay of Bengal where oxygen and O_2_* in this model have unrealistic values^62^.

### Model anthropogenic ΔCant’

The component ΔCant’ was derived from Eq. (S1) (ΔCant’ = ΔCO_2_ − ΔCant_0_ − ΔCO_2Climate_) using CMIP5 model simulations. ΔCO_2_ was taken from experiment RCP8.5, ΔCant_0_ from experiment esmFixClim3, and ΔCO_2Climate_ from experiment esmFdbk3. Note that the control simulation was also used to correct model drift. We estimated ΔCant’ = 0.05 ± 0.05 PgC yr^−1^ for the 1991 to 2016, based on the results of the three models, which individually yields ΔCant’ of 0.0 PgC yr^−1^ (IPSL), 0.11 PgC yr^−1^ (GFDL) and 0.11 PgC yr^−1^ (UVic), and assuming an uncertainty of ±100%. This corresponds to a trend of 0.11 ± 0.11 per meg yr^−1^.

### Model ΔAPO_Climate_ to ΔOHC ratios and uncertainty

Model ΔAPO_Climate_ is computed using individual contributions from O_2_, CO_2_ and N_2_ via:

$$\begin{array}{c}{\Delta {\rm{APO}}}_{{\rm{Climate}}}[{\rm{per}}\,{\rm{meg}}]={\Delta {\rm{APO}}}_{({\rm{O}}2)}+{\Delta {\rm{APO}}}_{({\rm{CO}}2)}+{\Delta {\rm{APO}}}_{({\rm{N}}2)}\\ {\Delta {\rm{APO}}}_{{\rm{Climate}}}[{\rm{per}}\,{\rm{meg}}]=\frac{1}{{{\rm{X}}}_{{\rm{O}}2}\,{{\rm{M}}}_{{\rm{air}}}}\times ({\Delta {\rm{F}}}_{{\rm{O}}2}+{{\rm{\alpha }}}_{{\rm{B}}}\times {\Delta {\rm{F}}}_{{\rm{CO}}2}-\frac{{{\rm{X}}}_{{\rm{O}}2}}{{{\rm{X}}}_{{\rm{N}}2}}\times {\Delta {\rm{F}}}_{{\rm{N}}2})\end{array}$$

where ΔF_O2_, ΔF_CO2_ and ΔF_N2_ are the changes in air-sea fluxes of O_2_, CO_2_ and N_2_ respectively (in moles), M_air_ is the number of moles of dry air in the atmosphere and X_N2_ and X_O2_ are the reference atmospheric mixing ratio of N_2_ and O_2_ respectively^63^. O_2_ and CO_2_ fluxes are simulated in the models. N_2_ air-sea fluxes, which impact the O_2_ atmospheric mixing ratio (because O_2_ is ~20% of the atmospheric composition), are quantified from the global ocean temporal changes in N_2_ solubility computed from model changes in temperature and salinity^64^.

The link between long-term changes in APO_Climate_ and ocean heat content, i.e. ΔAPO_Climate_-to-ΔOHC ratios, were computed for each model using the 180 years of simulations (1920 to 2100). Resulting ΔAPO_Climate_-to-ΔOHC ratios vary between 0.82 and 0.98 per meg per 10^22^ J of warming (Fig. S3). These ratios include uncertainty in the natural climate variations at interannual and decadal time-scales and uncertainty in the O_2_:C oxidative ratio associated with global gains and losses of O_2_ and CO_2_ by terrestrial ecosystems (α_B_ = 1.05 ± 0.05). The uncertainty due to interannual variations was evaluated by computing ΔAPO_Climate_-to-ΔOHC ratios using multiple 26 year-long segments from the 180 year simulations. We obtained 616 ΔAPO_Climate_-to-ΔOHC ratios (154 time-series of 26 years per model) and used the standard deviation between these ratios as a measure of the uncertainty.

We combine results from all models along with modeled and observed hydrographic relationships between OPO and potential temperature to establish an observationally-constrained estimate of the ΔAPO_Climate_-to-ΔOHC ratio, as shown in Fig. S3b. We first carry out this procedure with a fixed oxidative ratio α_B_ of 1.05, which yields ΔAPOClimate-to-ΔOHC = 0.86 ± 0.03 per mer per 10^22^ J. Repeating the same procedure using oxidative ratios α_B_ of 1.0 and 1.1 applied to both APO and OPO yield differences in ΔAPO_Climate_-to-ΔOHC smaller than 0.01 per meg per 10^22^J, showing that sensitivity to the oxidative ratio is very small. Note that on Fig. S3b, the model error bars include two contributions to the uncertainties on the simulated ΔAPO_Climate_-to-ΔOHC ratios (interannual variations and O_2_:C ratio) that combine to yield ±0.01 per meg per 10^22^ J for the CESM and GFDL models, ±0.02 per meg per 10^22^ J for the UVic model and ±0.05 per meg per 10^22^ J for the IPSL model (1-sigma).

### Steric component of sea level rise

We evaluated the steric component of sea level rise associated with a OHC of 1.3 × 10^22^ J yr^−1^ to be 1.53 mm y^−1^. Following ref. ^65^, this calculation assumes that 45% of the warming occurs below 700 m and that the steric rise is 1 mm per 0.60 × 10^22^ J above 700 m and 1 mm per 1.15 × 10^22^ J below 700 m (i.e. global steric rise of 1 mm per 0.84 × 10^22^ J). Assuming that 48% of the warming occurs below 700 m (ref. ^6^) would yield a global steric rise of 1 mm per 0.86 × 10^22^ J and change our estimate by less than 3%. Our estimate is also consistent with the recent hydrography-based estimate of the WCRP Global Sea Level Budget Group^66^.

### Link to global ocean de-oxygenation

Our application of O_2_ atmospheric measurements to constrain long-term ocean warming can be compared with earlier work considering warming-driven oceanic O_2_ outgassing. Multiplying our warming rate of 1.29 ± 0.79 × 10^22^ J yr^−1^ by the O_2_-to-heat ratios simulated by the four ESMs (−3.70 ± 0.80 nmol O_2_ J^−1^), yields an ocean loss of 48 ± 30 Tmol O_2_ y^−1^. Adding a loss of ~19 Tmol O_2_ y^−1^ due to anthropogenic aerosols (Table S5), yields a global ocean outgassing of 67 ± 35 Tmol O_2_ y^−1^, in the range of previous estimates based on atmospheric data^67^ (~40 Tmol O_2_ y^−1^), ocean data above 1000 m^68,69^ (55–65 Tmol O_2_ y^−1^) and global ocean data^70^ (96 ± 42 Tmol O_2_ y^−1^). This calculation implies that ocean CO_2_ uptake is reduced by warming at a ratio of ~0.70 nmol of CO_2_ per Joule (difference between O_2_-to-heat ratio of 3.70 nmol J^−1^ and OPO-to-heat ratio of 4.43 nmol J^−1^).

## Supplementary information

Supplementary Information

## Data Availability

Scripps APO data are available at scrippso2.ucsd.edu/apo-data. Model results are available upon reasonable request to R. W. (IPSL anthropogenic aerosol simulations), L. B. (IPSL-CM5A-LR), M. C. L. (CESM-LE), J. P. D. (GFDL-ESM2M) and W. K. (UVic).

## Code availability

The code used to compute APO_Climate_ (10.5281/zenodo.2571986) is available at https://zenodo.org/record/2571986#.XQ1FYZNKhBw. Earth-system model codes are available online for IPSL-CM5A-LR (cmc.ipsl.fr/ipsl-climate-models), GFDL-ESM2M (mdl-mom5.herokuapp.com/web/docs/project/quickstart), UVic (climate.uvic.ca/model) and CESM (cesm.ucar.edu/models/).
