# Supplementary material for: Quantification of ocean heat uptake from changes in atmospheric O2 and CO2 composition
Source: Sci Rep. 2019 Dec 27;9:20244. doi: 10.1038/s41598-019-56490-z (PMC6934503; doi:10.1038/s41598-019-56490-z)
Supplement: Supplementary file 1 — Supplementary Information [file 41598_2019_56490_MOESM1_ESM.pdf]

Supplementary Information for

Quantification of Ocean Heat Uptake from Changes in Atmospheric O_2 and CO_2 Composition

L. Resplandy[1]*, R. Keeling[2], Y. Eddebar[2], M. Brooks[2], R. Wang[3], L. Bopp[3], M. C. Long[4], J. P. Dunne[5], W. Koeve[6], A. Oschlies[6]

[1]Department of Geosciences and Princeton Environmental Institute, Princeton University, Princeton, USA.

[2]Scripps Institution of Oceanography, University of California San Diego, La Jolla, USA.

[3]Department of Environmental Science and Engineering, Fudan University, Shanghai, 200433 China.

[4]Laboratoire de Météorologie Dynamique / Institut Pierre Simon Laplace, CNRS / ENS / X / UPMC, Département de Géosciences, Ecole Normale Supérieure, Paris.

[5]National Center for Atmospheric Research, Boulder, USA.

[6]NOAA, Geophysical Fluid Dynamics Laboratory, Princeton, USA.

[7]GEOMAR Helmholtz Centre for Ocean Research, Kiel, Germany.

*Correspondence to: laurer@princeton.edu.

This PDF file includes:

Figs. S1 to S4
Tables S1 to S6

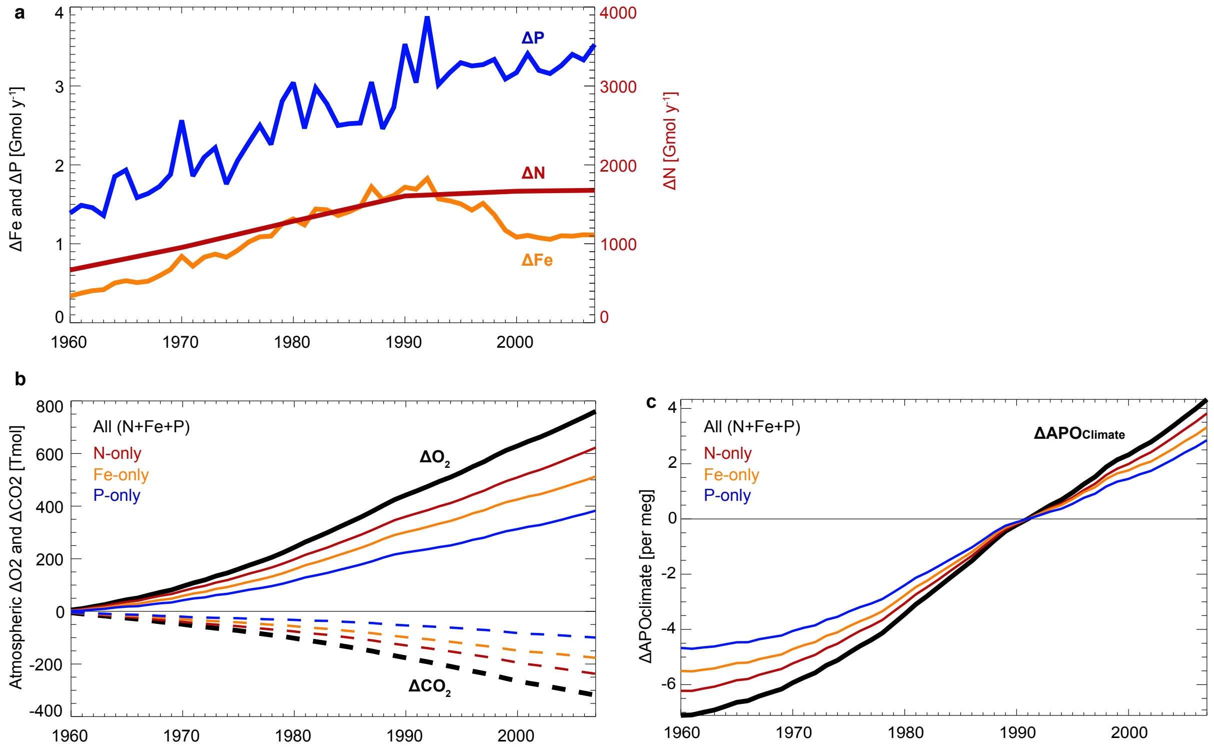

Figure S1. Effects of anthropogenic aerosols on APO. a. Anomaly, relative to 1850 levels, in deposition of atmospheric anthropogenic aerosols (N, P and Fe) at the air–sea interface between 1960 and 2007, derived from model simulations with and without aerosols [23]. b. Impact of aerosol eutrophication on atmospheric O_2 (solid lines) and CO_2 (dashed lines) for all aerosols (black lines) and for each aerosol taken individually (coloured lines). c, Overall impact of aerosol eutrophication on $\Delta APO_{Climate}$ referenced to the first year that has observations (1991).

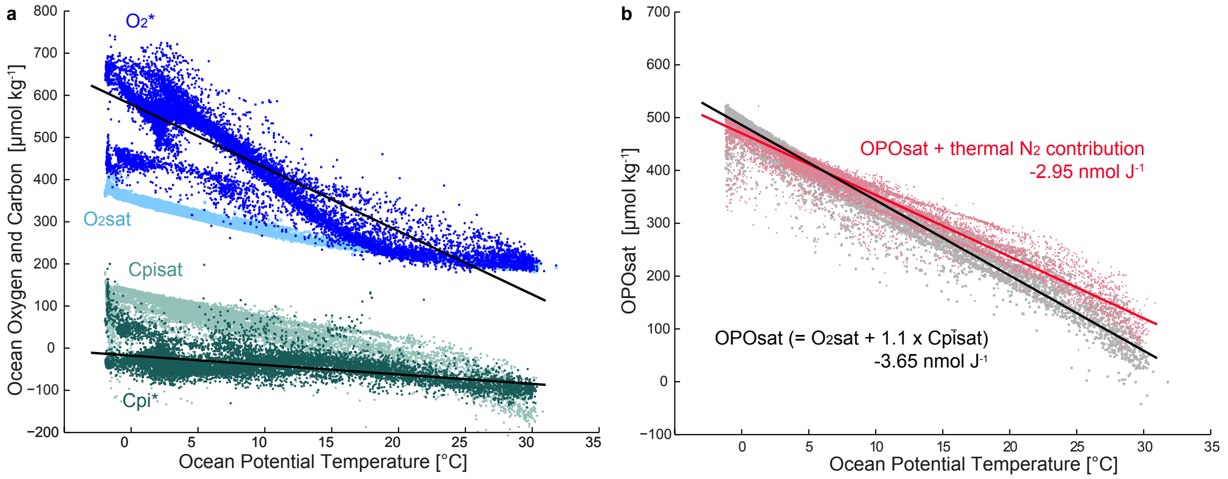

Figure S2. Solubility-driven changes in ocean oxygen and carbon concentrations. a. Ocean observations of O_2^*, O_2sat, C_{pi}^* and C_{pisat} as a function of potential temperature in the GLODAPv2 database [49]. b. OPO $_{sat}$ (= O2$_{sat}$ + 1.05 C_{pisat}, in grey) and the expected effects on APO owing to the combined effects of OPO $_{sat}$ and the thermal exchanges of N_2 (= O_{2sat} + 1.05 C_{pisat} – X_{O2} / X_{N2} [N_2 – mean(N_2)], in red). For clarity only 16 $\times 10^3$ points randomly picked out of the 78,456 data points available are shown for each variable. Note that very low values of O_2^* (around 450 µmol kg^{-1}) at low temperature (less than 10 °C) correspond to data collected in the Arctic Ocean, where phosphate concentrations (used for O_2^* calculation) are comparatively lower than in other cold ocean regions. Low O_2^* values in the Arctic explain the relatively low values of OPO shown in Fig. S3a at temperatures below 10 °C.

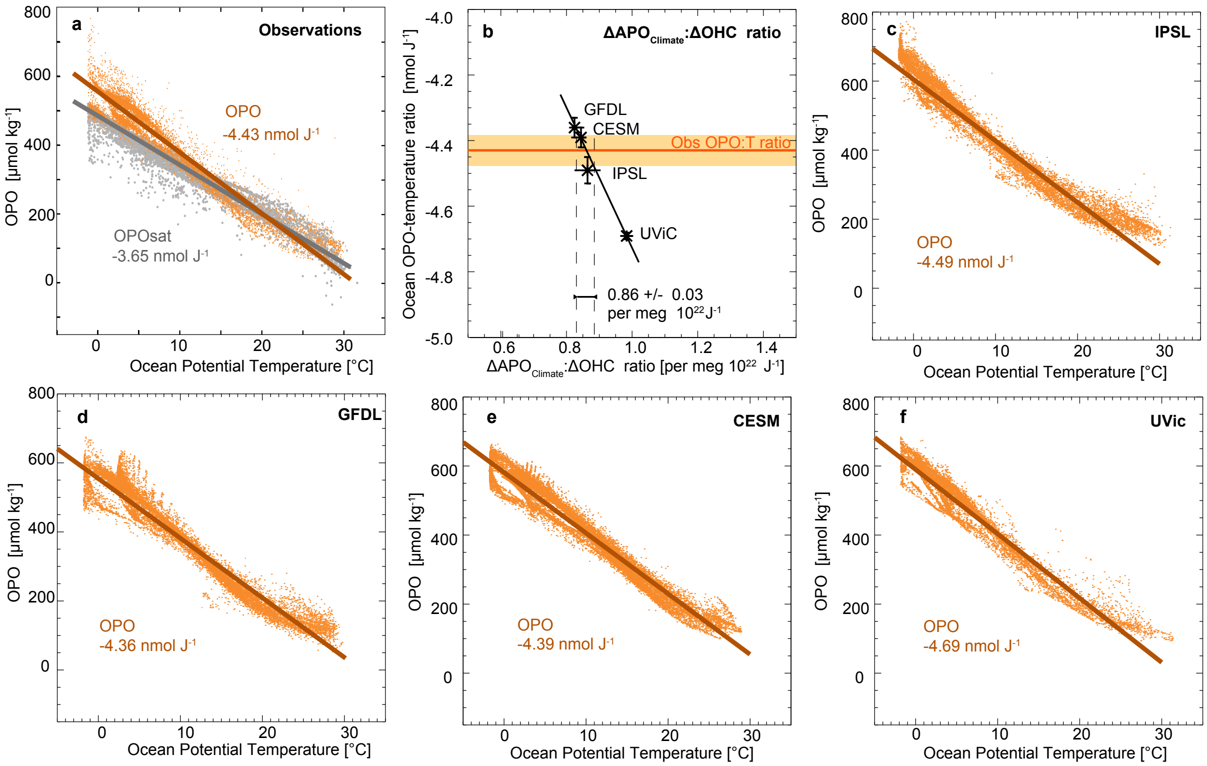

Figure S3. Link between OPO, $APO_{Climate}$ and ocean heat. a, c–f, OPO concentrations (yellow) and OPO concentrations at saturation based on O_2 and CO_2 solubility (OPO_{sat}, grey) as a function of ocean temperature in the GLODAPv2 database [49] (a) and four Earth-system models (IPSL, GFDL, CESM and UVic; c–f). Slopes give the OPO-to-temperature ratios in nmol J^{-1}. b. The link between $\Delta APO_{Climate}$ and changes in ocean heat content (that is, $\Delta APO_{Climate}$-to-ΔOHC ratio) in the four models is tied to their OPO-to-temperature ratios and can be constrained using the observed OPO-to-temperature of 4.43 nmol J−1 (vertical dashed lines). To avoid visual saturation, only 16,000 points, picked randomly, are shown for OPO.

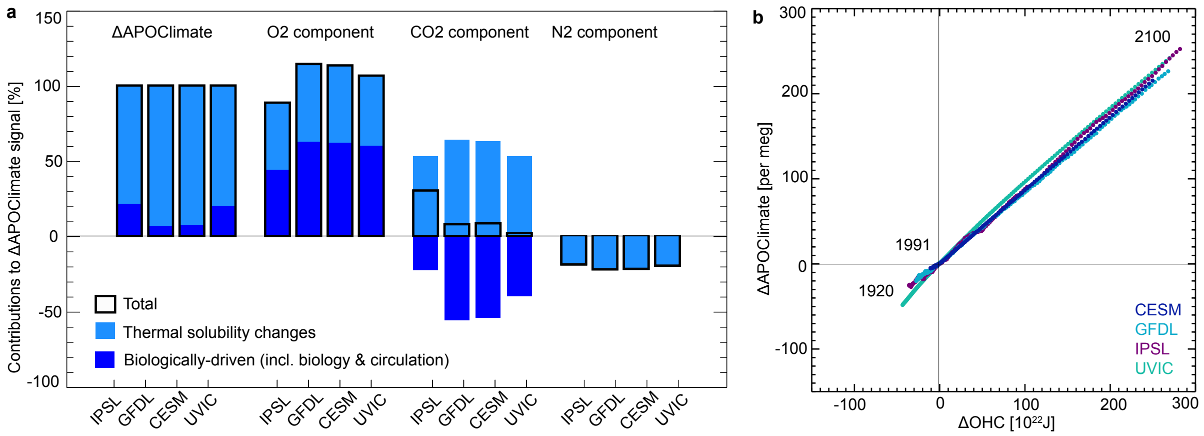

Figure S4. Changes in APOClimate ($\Delta APO_{Climate}$) and ocean heat content (ΔOHC) in four Earth-system models. A. Simulated $\Delta APO_{Climate}$ (black outlines) are decomposed into the contributions (percentage of total) from changes in ocean thermal saturation (light blue) and biologically driven changes (dark blue), the latter including changes in photosynthesis/respiration and changes in ocean circulation that transport and mix gradients of biological origin. For each model, ΔAPOClimate is further decomposed into its O_2, CO_2 and N_2 components—that is, how much of $\Delta APO_{Climate}$ is explained by changes in O_2, CO_2 and N_2 air–sea fluxes due to ocean saturation changes and biologically driven changes. b. Model $\Delta APO_{Climate}$-to-ΔOHC ratios over the 180 years of simulation (referenced to year 1991) in per meg per 10^{22} J units are: 0.85 ± 0.01 (CESM), 0.83 ± 0.01 (GFDL), 0.89 ± 0.03 (IPSL) and 0.99 ± 0.02 (UVic).

Table S1. Sources of the hydrographic databased estimates of global changes in ocean heat content (ΔOHC) used in Fig. 1.

**Label in Fig 1**	**0 to 2000 m depth range**	**2000 to 6000 m depth range**
PMEL	Ref. 6	Ref. 7
MRI	Ref. 5	Ref. 7
NCEI	Update of ref. 47	Ref. 7
CHEN	Ref. 8	Ref. 7

Table S2. Linear trends in ocean heat content.

	**1991-2016** OHC trend (±1σ)	**1993-2016** OHC trend (±1σ)	**2007-2016** OHC trend (±1σ)
$APO_{Climate}$	1.29 ± 0.79	-	-
PMEL	-	1.35 ± 0.10	1.16 ± 0.20
MRI	1.00 ± 0.11	1.03 ± 0.12	1.23 ± 0.22
NCEI	0.89 ± 0.08	0.90 ± 0.09	1.28 ± 0.16
CHEN	1.07 ± 0.07	1.10 ± 0.08	1.09 ± 0.10

Units are 10^{22} J yr^{-1}. Trends and ±1σ uncertainty ranges are given for hydrographic (in situ temperature) and atmospheric (APO) data over the depth range 0–6,000 m. See Table S1 for literature sources of estimates.

Table S3. Contributions to ΔAPO_{OBS}, ΔAPO_{FF} and ΔAPO_{Cant} and associated uncertainties (±1σ) during the observation period (1991 to 2016).

	Mean value	References	1σ uncertainty	References
		**ΔAPO_{OBS}**		
Corrosion			± 0.3 per meg yr^{-1}	
Leakage			± 0.2 per meg yr^{-1}	
Desorption			± 0.1 per meg yr^{-1}	
Thermal fractionation			± 2 per meg (± 4 before July 1992)	Ref. 35
Scale systematic error			2% on $\delta(O_2/N_2)$ contribution	
		**ΔAPO_{FF}**		
		**Oxidative Ratios R_i**		
Coal	1.17		± 0.03	
Oil	1.44	Ref. 18	± 0.03	Ref. 18[+]
Gas	1.95		± 0.04	
Cement	0.0		± 0.00	
Flaring	1.98		± 0.07	
		**Emissions $\Delta CO2_i$**		
Coal			± 7.0%	
Oil	Time varying	Ref. 21	± 5.5%	Ref. 36
Gas			± 6.5%	
Cement			±12%	
Flaring			±12%	
		**ΔAPO_{Cant}**		
**$\Delta Cant_0$**	Time varying (-2 to -3 PgC yr^{-1})	Ref. 22	1-σ of 10 experiments (<0.3 PgC yr^{-1})	Ref. 22
			+ 1% uncertainty (<0.03 PgC yr^{-1}) (atmospheric CO_2 history)	*this study*
**$\Delta Cant'$**	0.05 PgC yr^{-1} (0.11 per meg yr^{-1})	*this study*	± 0.05 PgC yr^{-1} (± 0.11 per meg yr^{-1})	*this study*
		**α_B**		
Terrestrial oxidative ratio α_B	1.05	Ref. 20	±0.05	Ref. 20

[+] Uncertainties in fossil-fuel oxidative ratios are ultimately from Keeling, 1988, Developing an interferometric oxygen analyzer for precise measurements of the atmospheric O_2 mole fraction, PhD thesis, Harvard University. We interpret these as 1-sigma uncertainties despite language in Keeling (1988) that suggest they may be 90% confidence intervals because they are based on 1-sigma spread across fuels.

Table S4. Temporal evolution of the cumulative contributions to global APO changes and their 1σ uncertainties.

year	ΔAPO$_{Climate}$	1σ	ΔAPO$_{OBS}$	1σ	ΔAPO$_{FF}$	1σ	ΔAPO$_{AtmD}$	1σ	ΔAPO$_{AtmD}$	1σ	Oxidative ratio 1σ
1991	0.00	0.00	0.00	0.00	0.00	0.00	0.00	0.00	0.00	0.00	0.00
1992	0.68	4.06	-8.43	4.02	-4.69	0.17	-4.71	0.51	0.28	0.14	0.14
1993	4.00	4.16	-14.14	4.09	-9.44	0.36	-9.26	0.57	0.56	0.28	0.28
1994	3.38	2.64	-24.16	2.42	-14.22	0.57	-14.16	0.65	0.84	0.42	0.42
1995	4.29	3.04	-32.94	2.73	-19.04	0.79	-19.30	0.76	1.12	0.56	0.56
1996	6.85	3.51	-40.28	3.09	-24.00	1.03	-24.53	0.88	1.40	0.70	0.70
1997	8.87	3.99	-48.03	3.46	-28.98	1.28	-29.60	1.00	1.68	0.84	0.84
1998	12.60	4.55	-54.77	3.91	-34.06	1.54	-35.26	1.14	1.96	0.98	0.98
1999	15.91	5.12	-61.88	4.36	-39.19	1.80	-40.84	1.28	2.24	1.12	1.12
2000	10.55	5.75	-77.63	4.88	-44.47	2.06	-46.23	1.41	2.52	1.26	1.26
2001	11.36	6.34	-87.34	5.36	-49.81	2.34	-51.68	1.55	2.80	1.40	1.40
2002	16.19	6.90	-93.32	5.79	-55.21	2.62	-57.37	1.70	3.08	1.54	1.54
2003	15.16	7.59	-105.69	6.36	-60.82	2.92	-63.39	1.85	3.36	1.68	1.68
2004	15.37	8.25	-116.92	6.89	-66.60	3.23	-69.32	1.99	3.64	1.82	1.82
2005	20.35	8.88	-123.73	7.39	-72.53	3.55	-75.47	2.14	3.92	1.96	1.96
2006	21.38	9.58	-134.69	7.95	-78.55	3.87	-81.71	2.29	4.20	2.10	2.10
2007	20.78	10.28	-147.25	8.52	-84.64	4.22	-87.88	2.44	4.48	2.24	2.24
2008	19.29	10.99	-160.91	9.08	-90.89	4.57	-94.06	2.59	4.76	2.38	2.38
2009	23.15	11.61	-169.09	9.54	-96.92	4.93	-100.36	2.74	5.04	2.52	2.52
2010	23.14	12.35	-181.69	10.14	-103.24	5.29	-106.90	2.89	5.32	2.66	2.66
2011	23.88	13.05	-193.54	10.68	-109.66	5.66	-113.35	3.04	5.60	2.80	2.80
2012	25.09	13.78	-205.11	11.27	-116.14	6.04	-119.93	3.17	5.88	2.94	2.94
2013	24.95	14.58	-218.28	11.93	-122.61	6.43	-126.77	3.30	6.16	3.08	3.08
2014	26.94	15.29	-229.40	12.49	-129.09	6.83	-133.67	3.43	6.44	3.22	3.22
2015	26.34	16.04	-243.32	13.08	-135.71	7.23	-140.66	3.56	6.72	3.36	3.36
2016	27.21	16.85	-255.93	13.74	-142.38	7.65	-147.75	3.69	7.00	3.50	3.50

Table S5. Trends in air–sea flux of O_2, CO_2 and APO due to anthropogenic aerosol deposition

Trends 1980 to 2007	N-only	Fe-only	P-only	All (N+Fe+P)
O_2 [Tmol y^{-1}]	15.5	12.9	9.6	19.0
CO_2 [Tmol y^{-1}]	-6.1	-4.5	-2.6	-8.3
$APO_{AtmD(O2)}$ [per meg yr^{-1}]	0.42	0.35	0.26	0.51
$APO_{AtmD(CO2)}$ [per meg yr^{-1}]	-0.17	-0.13	-0.07	-0.23
APO_{AtmD} [per meg yr^{-1}]	0.25	0.22	0.19	**0.28**

Table S6. Contributions to the uncertainty in $\Delta APO_{Climate}$ (1σ)

Source of uncertainty	Uncertainty in $\Delta APO_{Climate}$ trend (in per meg yr^{-1})
Measurement uncertainties	
Corrosion	0.30
Leakage	0.20
Desorption	0.10
Scale Error	0.39
Thermal Fractionation	0.06
Other uncertainties	
ΔAPO_{FF}	0.32
ΔAPO_{Cant}	0.14
ΔAPO_{AtmDep}	0.14
Land oxidative ratio α_B	0.14
Quadrature sum	0.68

Generated by isolating contributions to the 10^6-member ensemble, except the land oxidative ratio, which was estimated by differentiation of the central case where α_B=1.05 (see Methods).
